# Plasma Extracellular Vesicle Long RNA in Diagnosis and Prediction in Small Cell Lung Cancer

**DOI:** 10.3390/cancers14225493

**Published:** 2022-11-09

**Authors:** Chang Liu, Jinying Chen, Jiatao Liao, Yuchen Li, Hui Yu, Xinmin Zhao, Si Sun, Zhihuang Hu, Yao Zhang, Zhengfei Zhu, Min Fan, Shenglin Huang, Jialei Wang

**Affiliations:** 1 Department of Thoracic Medical Oncology, Fudan University Shanghai Cancer Center, and Shanghai Key Laboratory of Medical Epigenetics, International Co-laboratory of Medical Epigenetics and Metabolism, Institutes of Biomedical Sciences, Shanghai Medical College, Fudan University, Shanghai 200032, China; 2 Department of Oncology, Shanghai Medical College, Fudan University, Shanghai 200032, China; 3 Institute of Thoracic Oncology, Fudan University Shanghai Cancer Center, Shanghai 200032, China; 4 Department of Radiotherapy, Fudan University Shanghai Cancer Center, Shanghai 200032, China

**Keywords:** SCLC, extracellular vesicle, long RNA, t-signature

## Abstract

**Simple Summary:**

The present study firstly characterized the plasma exLRs profiles in SCLC patients and verified the feasibility and value of identifying biomarkers based on exLRs profiles in SCLC diagnosis and treatment prediction. We established a t-signature with good potency that can distinguish chemo-sensitive from chemo-refractory patients, which is conducive to precise individualized treatment. This signature also has potential clinical value for SCLC diagnosis, so that more patients can benefit from early diagnosis and optimal therapy.

**Abstract:**

(1) Introduction: The aim of this study was to identify the plasma extracellular vesicle (EV)-specific transcriptional profile in small-cell lung cancer (SCLC) and to explore the application value of plasma EV long RNA (exLR) in SCLC treatment prediction and diagnosis. (2) Methods: Plasma samples were collected from 57 SCLC treatment-naive patients, 104 non-small-cell lung cancer (NSCLC) patients and 59 healthy participants. The SCLC patients were divided into chemo-sensitive and chemo-refractory groups based on the therapeutic effects. The exLR profiles of the plasma samples were analyzed by high-throughput sequencing. Bioinformatics approaches were used to investigate the differentially expressed exLRs and their biofunctions. Finally, a t-signature was constructed using logistic regression for SCLC treatment prediction and diagnosis. (3) Results: We obtained 220 plasma exLRs profiles in all the participants. Totals of 5787 and 1207 differentially expressed exLRs were identified between SCLC/healthy controls, between the chemo-sensitive/chemo-refractory groups, respectively. Furthermore, we constructed a t-signature that comprised ten exLRs, including EPCAM, CCNE2, CDC6, KRT8, LAMB1, CALB2, STMN1, UCHL1, HOXB7 and CDCA7, for SCLC treatment prediction and diagnosis. The exLR t-score effectively distinguished the chemo-sensitive from the chemo-refractory group (*p* = 9.268 × 10^−9^) with an area under the receiver operating characteristic curve (AUC) of 0.9091 (95% CI: 0.837 to 0.9811) and distinguished SCLC from healthy controls (AUC: 0.9643; 95% CI: 0.9256–1) and NSCLC (AUC: 0.721; 95% CI: 0.6384–0.8036). (4) Conclusions: This study firstly characterized the plasma exLR profiles of SCLC patients and verified the feasibility and value of identifying biomarkers based on exLR profiles in SCLC diagnosis and treatment prediction.

## 1. Introduction

Lung cancer is the leading cause of cancer-related deaths worldwide [1]. Small-cell lung cancer (SCLC) is a high-grade neuroendocrine neoplasm accounting for approximately 15% of lung cancers [2,3]. Due to its rapid proliferation and potent aggressiveness, approximately 70% of SCLC patients are in the extensive stage (ES) at diagnosis [4]. For decades, the standard chemotherapy regimen used for SCLC is platinum-based chemotherapy combined with the etoposide chemotherapy drug [2]. Although highly sensitive to initial chemotherapy, more than 90% of patients eventually develop clinical drug resistance and die as a result of relapse [5]. However, to date, there are no clinically relevant screening methods or biomarkers to predict sensitivity to chemotherapy. Hence, there is an urgent need for defining biomarkers that could assist in risk assessment and stratification prior to the application of treatment. 

The genomic expression profiles of SCLC are difficult to obtain because of the limited SCLC cases receiving surgical resections, insufficient lung tissue biopsies, heterogeneity and low feasibility of secondary biopsies. Liquid biopsy represented by detecting circulating tumor DNA (ctDNA), circulating tumor cells (CTCs) and extracellular vesicles (EVs) is able to compensate for the above deficiencies and has presented significant roles in cancer diagnosis, prediction, prognosis and disease monitoring [6].

EVs, classified as exosomes and micro-vesicles, are nanometer-sized lipid bilayer structures that contain cell-derived proteins, lipids and various nucleic acids [7,8]. Secreted by living cells, EVs contains abundant biological information from their parental cells and can reflect the physiological and pathological states of a cell’s origin. In recent years, EV-derived long RNAs (exLR), including messenger RNA (mRNA), circular RNA (circRNA) and long non-coding RNA (lncRNA), have become a hotspot in cancer diagnosis, prediction and prognosis [9,10]. Zhao et al. observed that EV-derived lncRNA HOTTIP can be used as a diagnostic and prognostic marker for gastric cancer [11]. Yu, S. et al. characterized the plasma exLR profile in a pancreatic ductal adenocarcinoma and reported an exLR signature for the detection of pancreatic cancer [12]. Su, Y. et al. demonstrated the value of exLR profiling as potential biomarkers for the early detection and treatment efficacy prediction of breast cancer [13]. All these previous studies suggested that exLR may serve as a non-invasive diagnostic and prognostic biomarker. However, little research has focused on blood EV transcription profiles in SCLC. 

Our previous study established an optimized method for the exLR sequencing (exLR-seq) of human plasma [14] and identified exLR-based signatures to diagnose and predict the clinical outcomes of pancreatic ductal adenocarcinoma and breast cancer [12,15,16]. In this study, we characterize the exLR profile of 220 subjects, including 57 SCLC patients, 104 non-small-cell lung cancer (NSCLC) patients and 59 healthy participants, by RNA sequencing analyses; identify 10 plasma exLR biomarkers; and establish an exLR signature that can predict treatment sensitivity in SCLC patients, as well as separate SCLCs obtained from healthy participants and NSCLC patients. 

## 2. Materials and Methods

### 2.1. Participants and Research Design

The participants consisted of 57 SCLC patients, 104 NSCLC patients and 59 healthy participants receiving routine healthcare. All of the participants were recruited from the Fudan University Shanghai Cancer Center (FUSCC) between August 2018 and July 2021. All the SCLC patients were going to receive platinum-based chemotherapy (platinum in combination with etoposide or irinotecan) at FUSCC. None of the patients received any other forms of therapy during the time of enrollment. 

### 2.2. Assessments

Efficacy was assessed by progressive free survival (PFS), overall survival (OS), objective response rate (ORR) and disease control rate (DCR). PFS was defined as the time from the enrollment date until progressive disease (PD) occurrence or death from any cause. Patients alive without progression at the time of analysis were censored at their last follow-up session. OS was defined as the time from enrollment date to death due to any cause. Patients alive at the cutoff date were censored. DCR was defined as the percentage of patients with a complete response (CR), partial response (PR), or stable disease (SD). ORR was defined as the percentage of patients with CRs and PRs. The tumor response was assessed every two courses using the Response Evaluation Criteria in Solid Tumors (RECIST version 1.1).

SCLC patients who achieved CR or PR as the best response and had not progressed within the following 90 days after the end of first-line therapy were defined as chemo-sensitive. SCLC patients who had SD or PD as the best response or PD within the following 90 days after the termination of first-line therapy were defined as chemo-refractory [17,18].

### 2.3. Plasma Sample Collection

Blood samples were collected from all participants at baseline in 10 mL of EDTA-coated Vacutainer tubes. Among them, blood samples of 17 SCLC patients were also collected after 2 courses. The plasma was isolated by centrifugation at 800× *g* (~3000 rpm) for 10 min at room temperature (25 °C) within 2 h after blood collection and was then centrifuged at 16,000× *g* (~13,000 rpm) for 10 min at 4 °C to remove debris. Supernatants were aliquoted and stored at −80 °C prior to experimental processing and analysis.

### 2.4. Isolation and Characterization of EVs

The isolation of EVs was performed via the exoRNeasy Serum/Plasma Kit (Qiagen, Cat. No.77144, Hilden, Germany). Briefly, thawed plasma was mixed with binding buffer and added to the exoEasy membrane affinity spin column to bind the vesicles to the membrane. For transmission electron microscopy (TEM), size distribution measurements and Western blotting, the EVs were eluted with 400 μL of XE elution buffer (Qiagen, Cat. No. 76214). To reduce the eluate volume (to 50 μL), the samples were subjected to ultrafiltration using an Amicon Ultra-0.5 Centrifugal Filter 10 kDa (Merck Millipore, Cat No.UFC501008, Darmstadt, Germany). For EV RNA isolation, EVs were lysed on the column using QIAzol (Qiagen) and the total RNA was then eluted with 14 μL of RNase-free water.

#### 2.4.1. Transmission Electron Microscopy (TEM)

The EVs were identified by negative staining with phospho-tungstic acid. Ten microliters of resuspended EVs were placed on a parafilm membrane. A copper mesh with a formvar supporting membrane was covered with the EV suspension and floated for 3–10 min to allow for sample absorption into the supporting membrane. The fluid was then absorbed from the edges of the copper mesh with filter paper. Then, the copper mesh absorbing the sample was covered with 2% phospho-tungstic acid and floated for 3 min. The sample was dried for 10 min under incandescent light after the staining solution was absorbed with filter paper. Transmission electron micrographs were acquired using a transmission electron microscope (Phillips CM120, Tokyo, Japan) with a voltage of 120 kv.

#### 2.4.2. Size Distribution Measurement

The size distribution analysis of the EVs was performed on a Flow NanoAnalyzer (NanoFCM Inc., U30E, Xiamen, China), according to the manufacturer’s instructions. A series of monodisperse silica nanoparticles were synthesized and used as size reference standards to construct a calibration curve regarding particle sizes and side scattering intensities. Using this calibration curve, the size of every vesicle was determined.

#### 2.4.3. Western Blot Analysis

Peripheral blood mononuclear cells (PBMCs) were isolated by Lymphoprep (STEMCELL Technologies, Kent, WA, USA), according to the manufacturer’s instructions. PBMCs and EVs were lysed in RIPA buffer (1% NP40, 0.5% deoxycholate, 0.1% sodium dodecyl sulfate [SDS] in Tris-buffered saline) with protease inhibitors on ice for 30 min. Proteins obtained from EVs and PBMC were electrophoresed in 10% SDS-polyacrylamide gels and then transferred to 0.2 μm nitrocellulose membranes (Bio-Rad, Hercules, CA, USA). The membranes were blocked with 5% non-fat milk for one hour at room temperature. After probing with primary antibodies at 4 °C overnight, the membranes were incubated by HRP-conjugated secondary antibodies (Goat anti-Mouse IgG (H + L), Goat anti-Rabbit IgG (H + L), Proteintech). The antibodies used were anti-CD63 (Proteintech, 25682-1-AP), anti-TSG101 (Proteintech, 14497-1-AP) and anti-Calnexin (Proteintech, 10427-2-AP), goat anti-Mouse IgG (H + L) (Proteintech, SA00001-1) and goat anti-Rabbit IgG (H + L) (Proteintech, SA00001-2). The detection of immune complexes was performed using a LumiBest ECL Reagent Solution kit (Share-Bio, Shanghai, China).

### 2.5. RNA-Seq Analysis

Total EV RNA isolated from 1 mL of plasma was treated with DNase I (NEB, Cat. No. M0303S, Ipswich, MA, USA) to remove DNA. RNA-seq libraries were prepared using the SMARTer Stranded Total RNA-Seq Kit—Pico Input Mammalian (Clontech, Cat. No. 634414, Palo Alto, CA, USA). The library quality was estimated using the Qubit fluorometer (Thermo Fisher Scientific, Cat. No. Q33216, Waltham, MA, USA) and Qsep100 (BiOptic, New Taipei City, China). ExLR-seq was performed on an Illumina sequencing platform (San Diego, CA, USA) with 150 bp paired-end run metrics.

Raw reads were filtered using FastQC (version 0.11.8) and aligned to the GRCh38 human genome assembly using STAR (version 2.7.1a). Gene expression levels were calculated in transcripts per kilobase million (TPM). Annotations of mRNA and lncRNA in the human genome were retrieved from GENCODE (V.25). 

### 2.6. Data and Statistical Analyses

RNA-seq raw read counts were converted to TPM values to scale all comparable variates and normalized across all samples. Genes with frequencies of <25% were eliminated and the remaining exLRs were used for the subsequent analysis.

The transcriptional profiles of EVs obtained from the plasma were evaluated between different groups (SCLC vs. healthy controls, chemo-sensitive vs. chemo-refractory groups). The limma R package was used to identify the differential expression exLRs and the *p*-value of each marker was adjusted by the Benjamini–Hochberg method to control the false discovery rate (FDR). Gene ontology (GO) functional analysis and gene set enrichment analysis (GSEA) were performed to discover the pathway enrichment and significant molecular mechanisms of the different groups by using the ‘clusterProfiler’ package. The significant pathway was screened when the *p*-value < 0.05.

To identify the exLRs for SCLC treatment prediction and diagnosis, we used a multi-step approach. First, the exLRs differentially expressed between the chemo-sensitive and chemo-refractory groups were identified (*p* < 0.05). The analyses of exLRs associated with PFS and OS were also conducted using a Cox proportional hazards regression model (*p* < 0.05) and genes correlated with both PFS and OS were conserved to obtain survival-related genes. Meanwhile, we compared the exLRs between SCLC and healthy controls and this comparison discovered the genes that were specifically upregulated in SCLC (FDR < 0.05, fold change >2). Then, we intersected the genes to obtain the shared exLRs, through which ten exLRs were selected and logistic regression was used to train a signature (named as the t-signature) on the basis of the expression of exLR of SCLC patients (chemo-sensitive vs. chemo-refractory group). To assess the probability of prediction sensitivity to chemotherapy, we used the R function ‘predict’ to evaluate the prediction strength in quantitative terms in SCLC samples. The predictive efficacy of the t-signature was evaluated by receiver operating characteristic (ROC) curve analysis. Youden’s index was determined to identify the optimal cutoff point for calculating the exact predictive indices. The t-signature distribution in the different patient groups was tested by the Wilcoxon rank-sum test. The Kaplan–Meier curve and log-rank test were used to compare the survival rates of patients in the low- and high-value groups allocated by the t-signature. Cox regression was used to assess the t-signature’s performance for treatment prediction. Additionally, the t-signature was further validated for its diagnostic value in healthy controls and NSCLC patients.

All the statistical analyses were two-sided and a *p*-value <0.05 was considered statistically significant. The following R software packages were used in this study: ‘caret’, ‘enrichplot’, ‘glmnet’, ‘ggplot2′, ‘ggrepel’, ‘reshape2′, ‘survival’, ‘survminer’, ‘pROC’ and ‘pheatmap’. 

## 3. Results

### 3.1. Patients’ Characteristics and Treatment Patterns

In total, 220 individuals were included in this study, including 57 SCLC patients, 104 NSCLC patients and 59 healthy donors. Among the 57 SCLC patients, 25 (43.9%, 25/57) received the cisplatin plus etoposide (EP) regimen, whereas 31 (54.4%, 31/57) received carboplatin plus etoposide (EC) chemotherapy and one patient (1.8%, 1/57) received irinotecan plus cisplatin (IP). As defined above, 33 patients (33/57, 57.9%) were defined as chemo-sensitive and 24 patients (24/57, 42.1%) were chemo-refractory. The proportion of limited stage patients was higher in the chemo-sensitive cohort than that in the chemo-refractory cohort (*p* = 0.0057). Additionally, beyond that, there was no statistical difference between the two cohorts in other characteristics. The clinical features, including the age, stage and chemotherapy regimen of SCLC samples, are presented in Table 1.

### 3.2. Effectiveness Assessment

The tumor responses are presented in Table 2. A total of 42 patients (73.7%, 42/57) achieved PR, 11 (19.3%) had SD and four (7.0%) PD as the best response, resulting in an ORR of 73.7% (95% CI, 60.3–84.5%) and a DCR of 93.0% (95% CI, 83.0–98.1%). In the chemo-sensitive group (n = 33), 33 patients (100.0%) achieved PR, resulting in an ORR of 100.0% and a DCR of 100.0%. In the chemo-refractory group (*n* = 24), nine patients (37.5%) achieved PR. Eleven patients (45.8%) had SD and four patients (16.7%) reported PD as the best responses, resulting in an ORR of 37.5% and a DCR of 83.3%.

By the cutoff day (31 January 2021), 20 (66.7%) patients had developed disease progression. The estimated median PFS was 7.47 months (95% CI, 5.36 to 9.57 months) (Figure S1a). The estimated median OS was 22.03 months (95% CI, 17.65 to 26.41 months) (Figure S1b).

### 3.3. EV Isolation and Plasma exLR-Seq Analysis 

**Figure 1:**
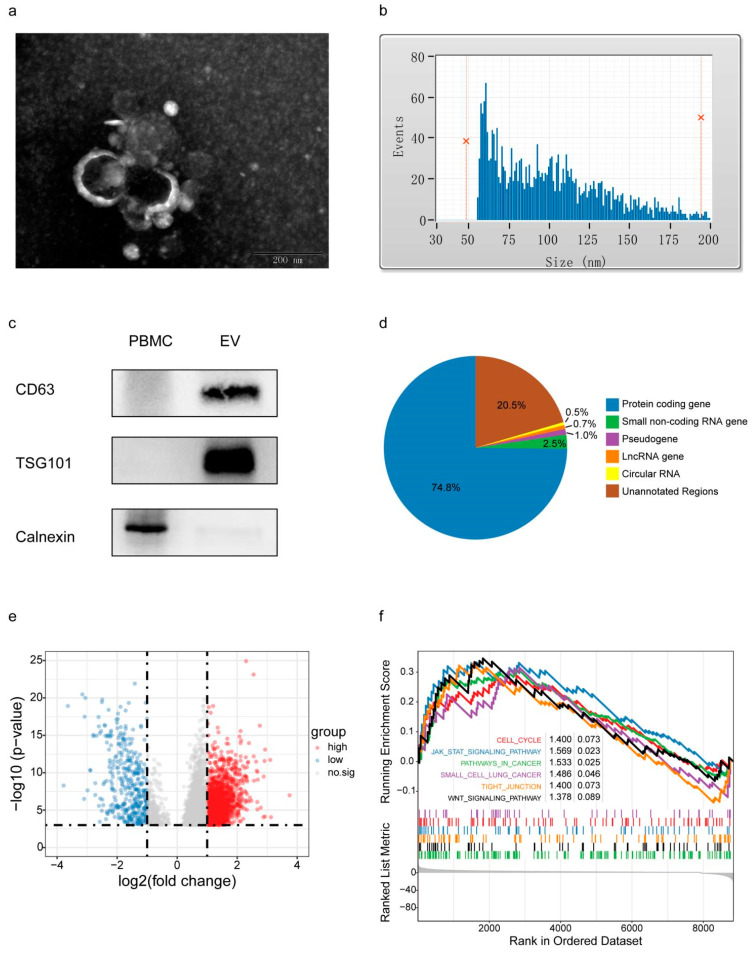
Plasma EV confirmation and exLR-seq analysis. (**a**) Transmission electron microscopy image of isolated vesicles, scale 200 nm. (**b**) Particle size analysis of isolated vesicles. (**c**) Western blots of calnexin, which should be detected in PBMC, but not in isolated vesicles, used as controls. EV markers TSG101 and CD63 detected in isolated vesicles, but not in PBMC. (**d**) Distribution of mapped reads to the annotated genes and identified circRNAs. (**e**) Volcano plot of differential expressed exLRs between SCLC and healthy controls. (**f**) The KEGG pathway enriched in the SCLC group by GSEA. EV: extracellular vesicle; PBMC: peripheral blood mononuclear cell; exLR: EV long RNA; SCLC: small-cell lung cancer; GSEA: gene set-enrichment analysis.

Plasma EVs were confirmed by TEM, size distribution measurement and Western blot analysis. As shown in Figure 1a, TEM exhibited the presence of rounded, cup-shaped, double-membrane-bound vesicle-like structures in the plasma. Moreover, flow cytometry suggested the presence of a heterogeneous population of spherical nanoparticles, with abundant peaks ranging from 50 to 200 nm and a mean diameter of 94.50 nm (Figure 1b). In addition, Western blot analysis showed that EV markers TSG101 and CD63 were enriched in these isolated vesicles but not in the peripheral blood mononuclear cell (PBMC), whereas calnexin (negative-control protein markers for EV identification) was detected in PBMC but not in isolated vesicles (Figure 1c; Figure S2: Original Western blot figures of Figure 1c).

We analyzed the exLR-seq results of plasma samples obtained from 57 SCLC patients (57 baseline samples plus 17 samples after two courses of chemotherapy), 104 NSCLC patients and 59 healthy controls. In the SCLC patients, NSCLC patients and healthy controls, approximately 20,000, 16,000 and 17,000 annotated genes, including mRNAs, lncRNAs and pseudogenes, were reliably detected, respectively. Protein-coding RNA (mRNA) constituted 74.8% of total mapped reads. Other RNA types accounted for a small fraction: 1.0% were pseudogenes, whereas lncRNAs and circRNAs were only 0.7% and 0.5%, respectively (Figure 1d).

#### Analysis of exLRs of SCLC-Group Patients and Control-Group Individuals

Firstly, we compared the gene expression profiles between the SCLC group and healthy controls. We identified 5787 exLRs that were differentially expressed in SCLC compared with controls (FDR < 0.001, (fold change) >2). Among them, 2053 were up-regulated in the SCLC group. The volcano map (Figure 1e) was drawn with differential expressed exLRs.

To explore the pathways associated with the differentially expressed exLRs, GSEA was conducted. The KEGG gene sets were chosen to perform the KEGG pathway-enrichment analysis among the SCLC group and healthy controls. Cancer-related pathways, such as cell cycle, JAK-STAT signaling pathway, pathways in cancer, small-cell lung cancer, tight junction and WNT signaling pathway, were enriched in the SCLC groups (*p* < 0.1) (Figure 1f). These results suggest that exLRs have potential as biomarkers for SCLC diagnosis and prediction.

### 3.4. Analysis of exLRs of SCLC Chemo-Sensitive and Chemo-Refractory Groups

**Figure 2:**
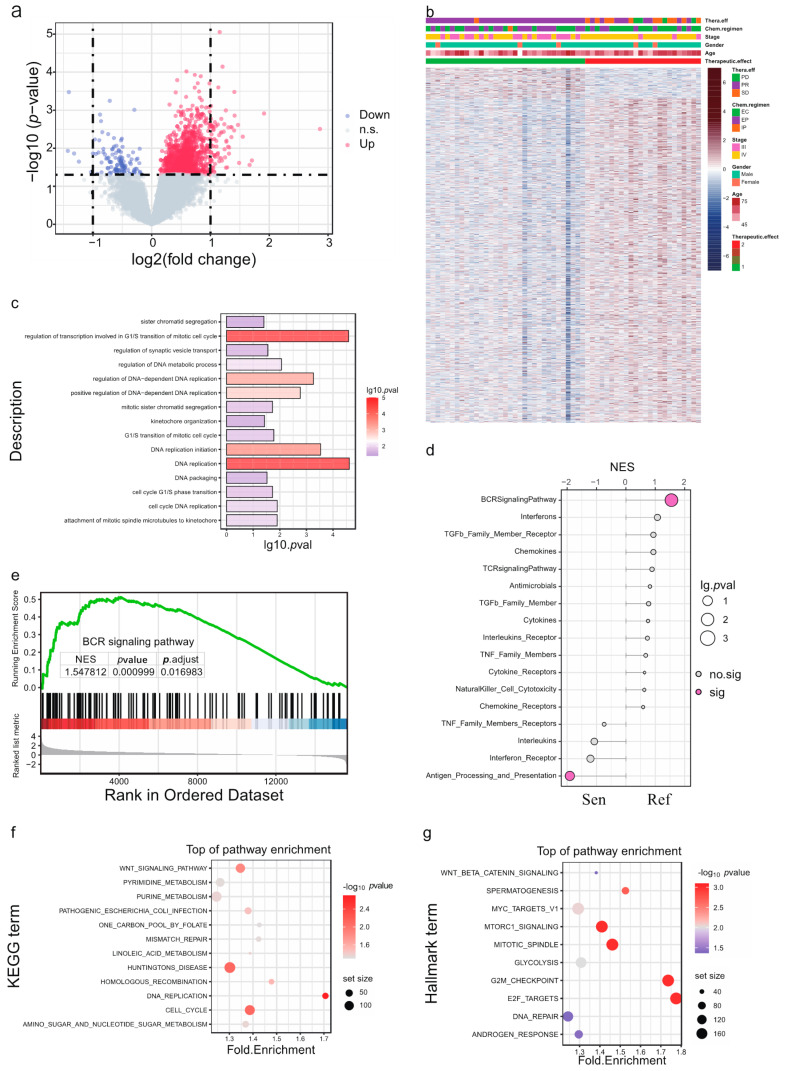
ExLR-seq analysis of patients with SCLC. (**a**) Volcano plot and (**b**) hierarchical clustering of differential expressed exLRs between chemo-sensitive and chemo-refractory groups. (**c**) GO analysis of the differentially expressed exLRs. (**d**) Enrichment score comparisons for immune-related pathways. (**e**) BCR signaling pathway enriched in the chemo-refractory group by gene set. (**f**) KEGG pathway analysis. (**g**) Enrichment score comparisons for hallmark analysis. GO: gene ontology; KEGG: Kyoto Encyclopedia of Genes and Genomes.

We also used R-package “limma” to obtain differentially expressed exLRs based on a filter criterion of *p* < 0.05. A total of 1207 exLRs were identified between the chemo-sensitive and chemo-refractory groups; most were up-regulated in the chemo-refractory group. A volcano plot was used for visualizing the exLRs (Figure 2a). Additionally, the heatmap shows a clear separation between the chemo-sensitive and chemo-refractory groups (Figure 2b).

The GO enrichment was firstly performed based on these differentially expressed exLRs. As shown in Figure 2c, GO-enrichment analysis demonstrates that the exLRs are mainly involved in the biological processes connected with the G1/S transition of the mitotic cell cycle, DNA replication and DNA replication initiation. In order to explore the underlying pathway mechanism in the therapeutic effect, immune-related pathways, KEGG pathways and the hallmark analysis of differentially expressed exLRs using GSEA were then performed. The activities of most immune pathways in the chemo-refractory group were greater than those in the chemo-sensitive group (Figure 2d). In particular, as shown in Figure 2e, the BCR signaling pathway was notably enriched in the chemo-refractory group. Furthermore, the most significantly enriched gene sets positively correlated with the chemo-refractory group and many enriched KEGG pathways were closely related to cancer, such as DNA replication, cell cycle and the WNT signaling pathway (Figure 2f). Similarly, SCLC in the chemo-refractory group was enriched in cell proliferation-related gene sets, including E2F targets, G2M checkpoint, MYC targets v1 and MTORC1 signaling, as well as cancer stemness-related gene sets, Wnt beta-catenin signaling (Figure 2g).

### 3.5. Blood exLRs May Reflect the Fractions of Different Cell Types

**Figure 3:**
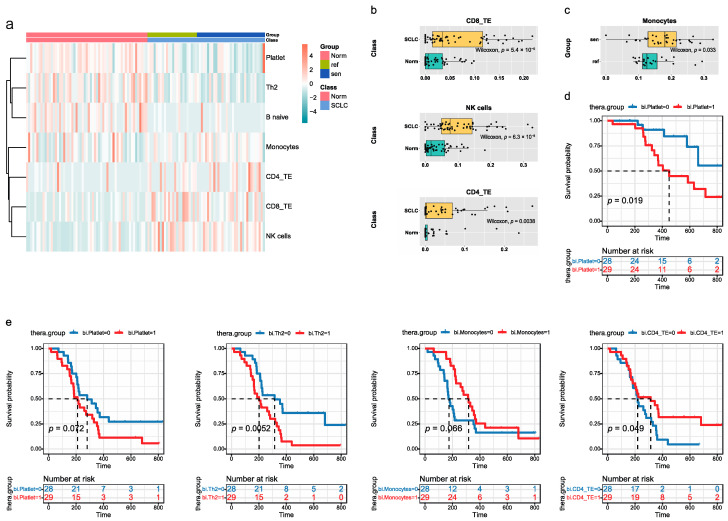
Comparison of different cell types from the exLR-seq data. (**a**) Heatmap of hierarchical clustering of cell types differentially observed in different groups. Boxplots of cell-type abundance between healthy controls and SCLC (**b**) and between chemo-refractory and chemo-sensitive groups (**c**). Prognostic significance of cell types by OS (**d**) and PFS (**e**). OS: overall survival; PFS: progressive free survival.

Since blood EVs are derived from a variety of tissues, we used EV-origin to characterize the source contribution of the cell fractions obtained from the exLR-seq profiles. EV-origin is a package that resolves the cellular origin from the plasma exLR gene expression data of 23 hemopoietic cells. Firstly, the components with frequencies of <10% (i.e., expressed in less than 10% of the entire samples) were omitted and the remaining 21 components were used for subsequent statistical analyses. As shown in Figure 3a, the cell component is estimated based on the gene expression profile and differences can be observed between the healthy controls vs. SCLC and the chemo-refractory vs. chemo-sensitive group. Among them, NK, CD8_TE and CD4_TE were significantly enriched in SCLC (*p* < 0.01, FC > 2), indicating the tumor-immune responses in the SCLC (Figure 3b). Meanwhile, a slight decrease in monocytes was observed in the chemo-refractory group compared with the chemo-sensitive group (*p* = 0.033) (Figure 3c). In addition, the survival analysis results show a negative correlation between OS and the abundance of platelets (Figure 3d). As shown in Figure 3e, patients in the group with a higher quantity of platelets and Th2 have shorter PFS rates, while longer PFS rates can be observed in patients with enriched monocytes and CD4_TE. Thus, these components were supposed to play a role in SCLC prognostic predictions.

### 3.6. Identification of Differentially Expressed long RNA Candidates and Model Construction

The different exLRs profiling between the groups mentioned above implies that the exLRs have potential as biomarkers for the stratification of SCLC. By intersecting differentially expressed exLRs related to PFS and OS, the chemo-sensitive vs. chemo-refractory group and specifics in the SCLC group compared to healthy controls, ten exLR markers (EPCAM, CCNE2, CDC6, KRT8, LAMB1, CALB2, STMN1, UCHL1, HOXB7 and CDCA7, as shown in Table 3) were selected and used to construct a SCLC classifier. The binary logistic regression analysis of the 10 exLRs was used to establish a predictive model and generated an exLR t-signature. Predictive scores (termed t-scores) of the samples were also obtained. 

#### Predictive Performance of exLR t-Score

**Figure 4:**
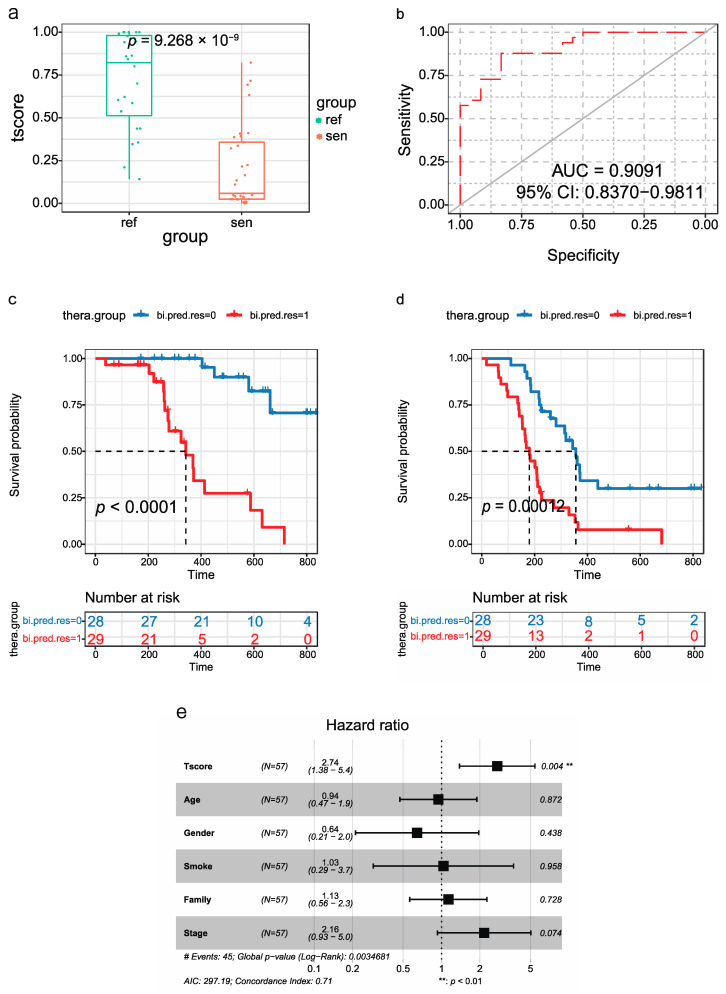
ExLR-based t-signature for the stratification of SCLC. (**a**) ExLR t-signature scores in chemo-sensitive (*N* = 33) and chemo-refractory (*N* = 24) groups. (**b**) ROC for the performance of the exLR t-signature in predictive chemotherapy treatment sensitivity of SCLC. (**c**,**d**) Kaplan–Meier survival analysis (log-rank test) of OS (**c**) and PFS (**d**) of SCLC patients with low (*n* = 28) or high (*n* = 29) t-scores. (**e**) Multivariate Cox proportional hazards regression analysis of clinical parameters and t-scores with PFS of SCLC patients. ROC: receiver operating characteristic.

According to the distribution of the t-scores and response status, the t-scores of chemo-refractory patients were notably higher than those of the chemo-sensitive group (*p* = 9.268 × 10^−9^) (Figure 4a). The t-signature differentiated the SCLC groups with an area under the receiver operating characteristic curve (AUC) of 0.9091(95%CI: 0.837–0.9811) (Figure 4b). These results demonstrate that we were able to successfully develop an EV-based pretreatment response prediction model in patients with SCLC.

Survival analyses were also performed on 57 SCLC patients applying the Kaplan–Meier Plotter tool. We utilized the median value of the t-scores as the boundary and separated the patients into high- (bi-pred.res = 1) and low-score (bi-pred.res = 0) groups. Patients in the high-score group had a significantly worse prognosis than the low-score group (*p*-value < 0.0001) (Figure 4c). Similarly, patients in the high-score group had a poorer progression-free survival (*p* = 0.00012) (Figure 4d). 

In order to validate the reliability of the signature from other perspectives, we analyzed the PFS with the t-score, age, gender, smoking history, family history of cancer and stage by multivariate Cox regression analyses. The t-score in the Cox analysis was highly significant (Figure 4e). Moreover, the t-score had the highest significance with a hazard ratio of 2.74, indicating that the t-score was a promising predictor independent of other clinico-pathological variables.

### 3.7. Analysis of t-Scores of 17 Paired Samples at Baseline and after Two Courses of Chemotherapy

**Figure 5:**
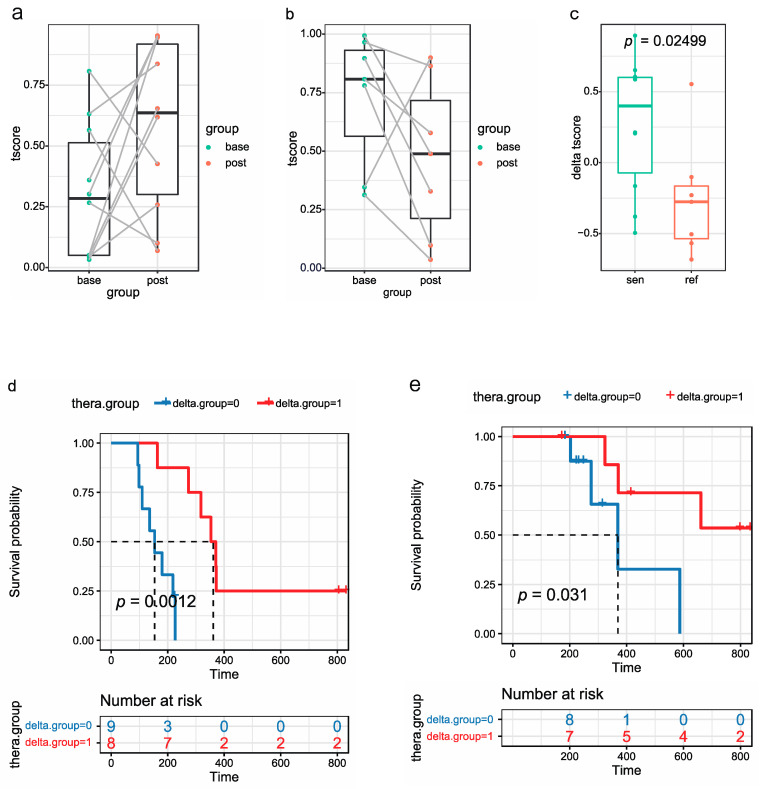
T-scores of paired samples at baseline and after 2 courses of chemotherapy. T-scores of chemo-sensitive (**a**) and chemo-refractory (**b**) groups at baseline and after 2 courses of chemotherapy. (**c**) T-score difference in chemo-sensitive and chemo-refractory groups. Kaplan–Meier survival analysis (log-rank test) of PFS (**d**) and OS (**e**) of SCLC patients with high and low t-score differences.

A major advantage of plasma exLR is the ease of availability of longitudinal samples to monitor tumor progression. To investigate whether the change in the t-score before and after the treatment could be used to predict treatment response, we analyzed the t-scores of 17 paired samples at baseline and following two courses of chemotherapy. The t-scores of 70% of samples (7/10) were upregulated after two courses of chemotherapy in the chemo-sensitive group and the t-scores of 85.7% of samples (6/7) were downregulated after two courses of chemotherapy in the chemo-refractory group (Figure 5a,b). Therefore, the t-score difference was analyzed further. The median value of the t-score difference in the chemo-sensitive group was greater than that in the chemo-refractory group (*p* = 0.02499) and may reflect the prognosis (Figure 5c). We utilized the median value of the t-score difference as the boundary and divided the patients into high- and low-value groups. The PFS and OS of the high-value group were both better than those of the low-value group (*p* = 0.0012; *p* = 0.031) (Figure 5d,e). 

### 3.8. Potential Diagnostic Values of exLR t-Signature

As 10 exLRs were enriched in SCLC samples compared with the healthy controls, we explored the diagnostic value of the exLR t-signature more extensively. The expression profile data of these 10 exLRs of the healthy controls and NSCLC samples were extracted and the t-scores were calculated. Then, we applied the ROC curve analysis to evaluate the diagnostic value of the signature.

**Figure 6:**
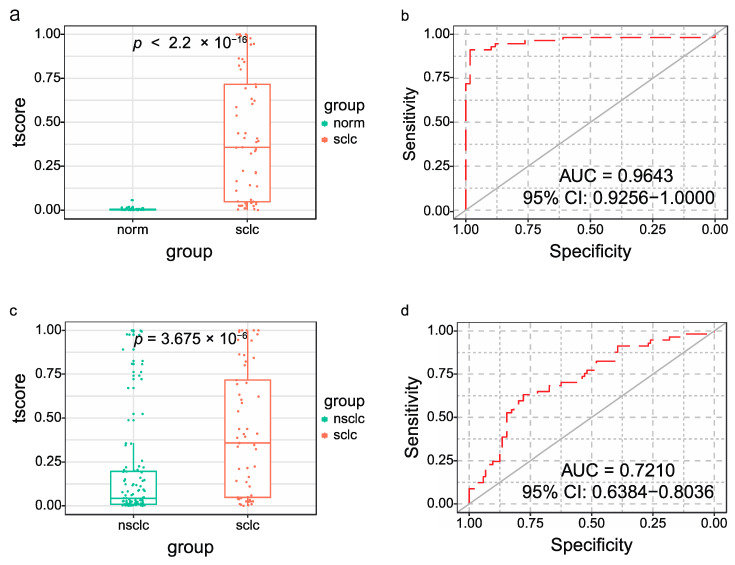
T-scores for the diagnosis of SCLC. T-scores in distinguishing SCLC from healthy controls (**a**) and SCLC from NSCLC (**c**). The ROC curve for the t-signature in SCLC and healthy controls (**b**), SCLC and NSCLC (**d**). NSCLC: non-small-cell lung cancer.

Compared with the healthy controls, the t-score was significantly higher in the SCLC group (*p* < 2.2 × 10^−16^) (Figure 6a). SCLC was detected with an AUC of 0.9643 (95% CI: 0.9256–1); the sensitivity and the specificity were 91.23% and 98.30%, respectively (Figure 6b). The diagnostic accuracy was also 94.83% (95% CI: 89.08% to 98.08%).

The t-signature was further applied to the NSCLC groups. As shown in Figure 6c, the t-score is also significantly higher in the SCLC group than that in the NSCLC group (*p* = 3.675 × 10^−6^). The t-signature distinguished SCLC from NSCLC patients with au AUC of 0.721 (95% CI: 0.6384–0.8036) (Figure 6d). The diagnostic accuracy was 72.67% (95% CI: 65.10% to 79.39%) with a sensitivity of 63.15% and a specificity of 77.88%. Additionally, the t-signature had high diagnostic values for SCLC from the healthy controls and NSCLC samples.

## 4. Discussion

In this study, we obtained exLR-seq expression profiles from 57 SCLC patients’ plasma EV samples (57 baseline samples plus 17 samples following two courses of chemotherapy), 104 NSCLC patients and 59 healthy individuals’ plasma EV samples, representing, to our knowledge, the largest and the first-published long RNA-seq expression profile library from SCLC plasma EVs. In addition, we, for the first time, identified the exLR profiles in SCLC and established a t-signature for SCLC prediction and diagnosis.

In this study, we firstly compared the differences in exLR levels between SCLC patients and healthy participants and exLRs enriched in SCLC were identified. Furthermore, we systematically explored the exLR between chemo-sensitive and chemo-refractory groups. To obtain greater insights into the functional roles of the differentially expressed exLRs in SCLC, we conducted the enrichment analysis of pathways, which presented several notably enriched pathway signals. Chemo-refractory groups were enriched in DNA replication, cell cycle, the WNT signaling pathway, E2F targets, G2M checkpoint, MYC targets v1 and mechanistic target of rapamycin complex 1 (MTORC1) signaling and Wnt beta-catenin signaling. Meanwhile, the cell component estimated based on the gene expression profile showed that natural killer (NK), terminal effector CD8 + (CD8_TE) and terminal effector CD4 + (CD4_TE) cells were enriched in the SCLC group and platelets, type-2 T-helper cells (Th2), CD4_TE and monocytes were related to the patient’s prognosis. Moreover, NK cells are cytotoxic innate lymphocytes that can kill tumor cells and are significantly different between normal and cancer tissues and the number of chimeric antigen receptor (CAR)-engineered NK (CAR-NK) preclinical studies is increasing year by year [18]. Terminally differentiated effector memory (TEMRA) cells are developed from long-lasting memory cells and studies show that higher frequencies of CD8_TE were observed in non-small-cell lung cancer (NSCLC) patients compared to healthy controls [19]. Willemsen A et al. revealed that the proportion of CD4+_TE cells was enriched in the responders and may be used to predict antitumor responses [20]. Liang P et al. analyzed the differential gene expression profiles in lung adenocarcinomas and observed that monocytes may suppress the progression of cancer; the upregulated expression of monocytes indicated a longer survival rate [21]. A platelet is a biomarker of disease burden and can affect the treatment outcomes of cancer patients. Ji Y et al. assessed the platelet count in 234 NSCLC patients and showed that the elevated preoperative platelet was associated with the poor prognosis of patients [22]. Xu et al. applied the immune profile analysis to patients and showed the upregulation of Th2 cells supported tumor growth and was associated with poor prognosis in hepatocellular carcinomas [23]. Further studies are required to explore the value of cell components estimated in SCLC diagnosis and prediction.

We performed the exLR profiling of plasma samples obtained from SCLC and healthy controls using an optimized exLR-seq strategy we recently developed [14]. Additionally, we selected ten exLR markers (EPCAM, CCNE2, CDC6, KRT8, LAMB1, CALB2, STMN1, UCHL1, HOXB7 and CDCA7) to construct a t-signature. For the specific ten markers identified in this paper, Gao P et al. observed that miR-3607-3p can inhibit NSCLC cell growth and metastasis by targeting TGFBR1 and CCNE2 protein expressions [24]. In addition, Wu D et al. observed that both CARM1 and CCNE2 were highly associated with shorter 10-year overall survival rates of at a large cohort of 461 cases of NSCLC patients and that CARM1 could promote NSCLC progression via the activation of CCNE2 [25]. In terms of CDC6, several studies revealed its correlation with prognosis in lung cancer. Allera-Moreau C et al. investigated that the expression of CDC6 was associated with overall, disease-free and relapse-free survival rates in early or mid-stage NSCLCs [26]. An C et al. also observed that CDC6 had a potential to be used as a circulating tumor cell biomarker for lung cancer by examining CDC6 expression from the PBMCs of patients with lung cancer [27]. Wang W et al. revealed that KRT8 was hypomethylated and overexpressed in lung adenocarcinomas and associated with unfavorable prognosis [28]. Additionally, several studies determined that UCHL1 may be a prognostic marker and therapeutic target in NSCLC patients [29,30]. In the field of EPCAM, many studies indicate that it can act as a potential diagnostic and prognostic biomarker for different types of cancers, including lung cancer [31,32,33]. Moin AT et al. suggested EPCAM to be a significant biomarker for human lung cancer prognosis [31]. Several studies verified that STMN1 was associated with worse survival rates for lung cancer patients [34,35,36,37]. Most of the ten markers were associated with the prognosis of lung cancer in previous research.

Most of the SCLC patients were diagnosed at the extensive stage and it was difficult to receive surgery. The establishment of predictive and diagnostic models is helpful for precise and individualized treatment. Here, we established a prognostic model and generated an exLR t-score for SCLC and we used this model to distinguish between chemo-sensitive and chemo-refractory patients with an AUC of 0.909 (95% CI: 0.837 to 0.981). The t-score has the potential to predict chemo-sensitive populations and is helpful for individualized precision treatment. We also scored the value of the model and divided the training into low and high t-score groups based on the median score. The prediction efficacy of the t-score was validated from several aspects. Firstly, ROC curves and Kaplan–Meier analysis were performed, which indicated that the t-signature had a good predictive value. A higher t-score was significantly associated with a poor disease outcome. Furthermore, the change in the t-score before and after the treatment was related to progressive disease and disease control, providing information for the prognosis for SCLC patients. Finally, the t-score can also be applied for SCLC diagnosis, which could effectively distinguish between the SCLC and healthy controls (AUC 0.9643, 95% CI: 0.9256-1), as well as differentiate between SCLC and NSCLC (AUC 0.721, 95% CI: 0.6384–0.8036). This predictive and diagnostic model facilitated the early diagnosis of SCLC and precise treatment as soon as possible.

This was the first study to characterize the exLR profile in SCLC patients and identify a t-signature with good potency, including in predicting the response to chemotherapy and diagnosis. There were some limitations to our study. Firstly, all the samples were obtained from the single center from the Fudan University Shanghai Cancer Center. In the future, we need to expand the sample size and collect samples from other centers as an independent validation cohort to validate the t-score model. Secondly, the model needs to be validated in other SCLC patients, such as those receiving immunotherapy as a first-line therapy. Thirdly, the cohort has a small number of female participants in the SCLC and healthy group as compared to male participants. We will delve into, in subsequent studies, whether this had an effect on the results.

## 5. Conclusions

In conclusion: our study indicated the value of exLR profiling in the diagnosis and prediction of SCLC and established a t-signature that can distinguish between chemo-sensitive and chemo-refractory patients, conducive to precise individualized treatment. This signature also has potential clinical value for SCLC diagnosis, so that more patients can benefit from early diagnosis and optimal therapy.

## Figures and Tables

**Table 1:** Baseline characteristics.

Characteristics	SCLC			Healthy Group (*n* = 59)
	Total (*n* = 57)	Chemo-Sensitive (*n* = 33)	Chemo-Refractory (*n* = 24)	
**Age, years**				
Mean	63.65	62.85	64.75	59.92
Median	64	62	66.5	58
Range	41–79	41–79	45–74	41–91
**Age group**				
<65 years	29 (50.9%)	19 (57.6%)	10 (41.7%)	40 (67.8%)
≥65 years	28 (49.1%)	14 (42.4%)	14 (58.3%)	19 (32.2%)
Sex				
Male	52 (91.2%)	30 (90.9%)	22 (91.7%)	47 (79.7%)
Female	5 (8.8%)	3 (9.1%)	2 (8.3%)	12 (20.3%)
**Smoking history**				
Never-smoker	6 (10.5%)	4 (12.1%)	2 (8.3%)	/
Former or current smoker	51 (89.5%)	29 (87.9%)	22 (91.7%)	/
**Family history of cancer**				
Yes	14 (24.6%)	10 (30.3%)	4 (16.7%)	/
No	43 (75.4%)	23 (69.7%)	20 (83.3%)	/
**ECOG PS at baseline**				
0	2 (3.5%)	2 (6.1%)	0 (0.0%)	/
1	54 (94.7%)	30 (90.9%)	24 (100.0%)	/
2	1 (1.8%)	1 (3.0%)	0 (0.0%)	/
**Stage**				
LS	20 (35.1%)	17 (51.5%)	3 (12.5%)	/
ES	37 (64.9%)	16 (48.5%)	21 (87.5%)	/
**Metastatic sites at baseline**				
Bilateral Lung	4 (7.0%)	1 (3.0%)	3 (12.5%)	/
Brain	10 (17.5%)	3 (9.1%)	7 (29.2%)	/
Bone	13 (22.8%)	3 (9.1%)	10 (41.7%)	/
Liver	10 (17.5%)	4 (12.1%)	6 (25.0%)	/
Adrenal gland	7 (12.3%)	3 (9.1%)	4 (16.7%)	/
Supraclavicular lymph node	8 (14.0%)	4 (12.1%)	4 (16.7%)	/
Pleural	16 (28.1%)	7 (21.2%)	9 (37.5%)	/
Others	18 (31.6%)	8 (24.2%)	10 (41.7%)	/
**Chemotherapy**				
EP	25 (43.9%)	18 (54.5%)	7 (29.2%)	/
EC	31 (54.4%)	14 (42.4%)	17 (70.8%)	/
IP	1 (1.8%)	1 (3.0%)	0 (0.0%)	/

EC: carboplatin plus etoposide; ECOG: Eastern Cooperative Oncology Group; ES: extensive stage; EP: cisplatin plus etoposide; IP: irinotecan plus cisplatin; LS: limited stage; PS: performance status; SCLC: small-cell lung cancer.

**Table 2:** Tumor responses.

Responses	Total (*n* = 57)	Chemo-Sensitive (*n* = 33)	Chemo-Refractory (*n* = 24)
CR	0 (0.0%)	0 (0.0%)	0 (0.0%)
PR	42 (73.7%)	33 (100.0%)	9 (37.5%)
SD	11 (19.3%)	0 (0.0%)	11 (45.8%)
PD	4 (7.0%)	0 (0.0%)	4 (16.7%)
ORR	73.7% [95% CI, 60.3–84.5%]	100.0% [95% CI, 89.4–100.0%]	37.5% [95% CI, 18.8–59.4%]
DCR	93.0% [95% CI, 83.0–98.1%]	100.0% [95% CI, 89.4–100.0%]	83.3% [95% CI, 62.6–95.3%]

CR, complete response; DCR, disease control rate; ORR, objective response rate; PD, progressive disease; PR, partial response; SD, stable disease.

**Table 3:** Characteristics of differential expressions of the 10 exLRs identified in this study.

exLRs	Chemo-Refractory vs. Chemo-Sensitive Group		SCLC vs. Healthy Controls		OS	PFS
	Mean Fold Change	*p*-Value	Mean Fold Change	FDR	*p*-Value	*p*-Value
CALB2	2.66	1.39 × 10^−3^	3.01	1.11 × 10^−6^	3.58 × 10^−4^	1.52 × 10^−6^
CCNE2	2.20	2.54 × 10^−4^	3.45	2.05 × 10^−11^	1.55 × 10^−5^	8.71 × 10^−7^
CDC6	1.59	1.12 × 10^−2^	2.69	3.85 × 10^−8^	1.36 × 10^−3^	1.36 × 10^−3^
CDCA7	1.90	8.60 × 10^−4^	2.02	1.54 × 10^−3^	1.70 × 10^−4^	1.34 × 10^−3^
EPCAM	2.28	3.83 × 10^−3^	4.51	2.12 × 10^−14^	1.50 × 10^−2^	5.68 × 10^−3^
HOXB7	1.62	4.31 × 10^−3^	2.46	6.71 × 10^−5^	3.87 × 10^−2^	9.71 × 10^−3^
KRT8	2.07	8.96 × 10^−3^	3.24	2.16 × 10^−7^	1.11 × 10^−2^	3.64 × 10^−3^
LAMB1	1.45	3.87 × 10^−2^	2.39	2.88 × 10^−7^	2.14 × 10^−2^	2.21 × 10^−2^
STMN1	1.56	1.77 × 10^−2^	2.07	7.37 × 10^−6^	4.43 × 10^−4^	1.75 × 10^−2^
UCHL1	2.36	2.34 × 10^−3^	2.21	4.70 × 10^−5^	4.32 × 10^−4^	1.76 × 10^−2^

## Data Availability

The datasets used and/or analysed during the current study are available from the corresponding author on reasonable request.

## Supplementary Materials

The following supporting information can be downloaded at: https://www.mdpi.com/article/10.3390/cancers14225493/s1, Figure S1: (a) Kaplan-Meier curve of progression free survival (PFS) of SCLC patients (N = 57). (b) Kaplan-Meier curve of overall survival (OS) of SCLC patients (N = 57). Figure S2: Original Western blot figures of Figure 1c.

## Abbreviations

AUC: area under the receiver operating characteristic curve; CR, complete response; DCR, disease control rate; EC, carboplatin plus etoposide; EP, cisplatin plus etoposide; ES, extensive stage; EV, extracellular vesicle; ExLR-seq, exLR sequencing; ExLR, extracellular vesicle long RNA; FDR, false discovery rate; GO, gene ontology; GSEA, gene set enrichment analysis; IP, irinotecan plus cisplatin; KEGG, Kyoto Encyclopedia of Genes and Genomes; NSCLC, non-small cell lung cancer; ORR, objective response rate; OS, overall survival; PBMC, peripheral blood mononuclear cell; PD, progressive disease; PFS, progressive free survival; PR, partial response; ROC, receiver operating characteristic curve; SD, stable disease; SCLC, small cell lung cancer; TPM, transcripts per kilobase million.
