# Supplementary material for: Plasma Extracellular Vesicle Long RNA in Diagnosis and Prediction in Small Cell Lung Cancer
Source: Cancers (Basel). 2022 Nov 9;14(22):5493. doi: 10.3390/cancers14225493 (PMC9688902; doi:10.3390/cancers14225493)
Supplement: Supplementary file 1 [file cancers-14-05493-s001.zip › cancers-1963990-supplementary.pdf]

cancers
MDPI

Supplementary Materials

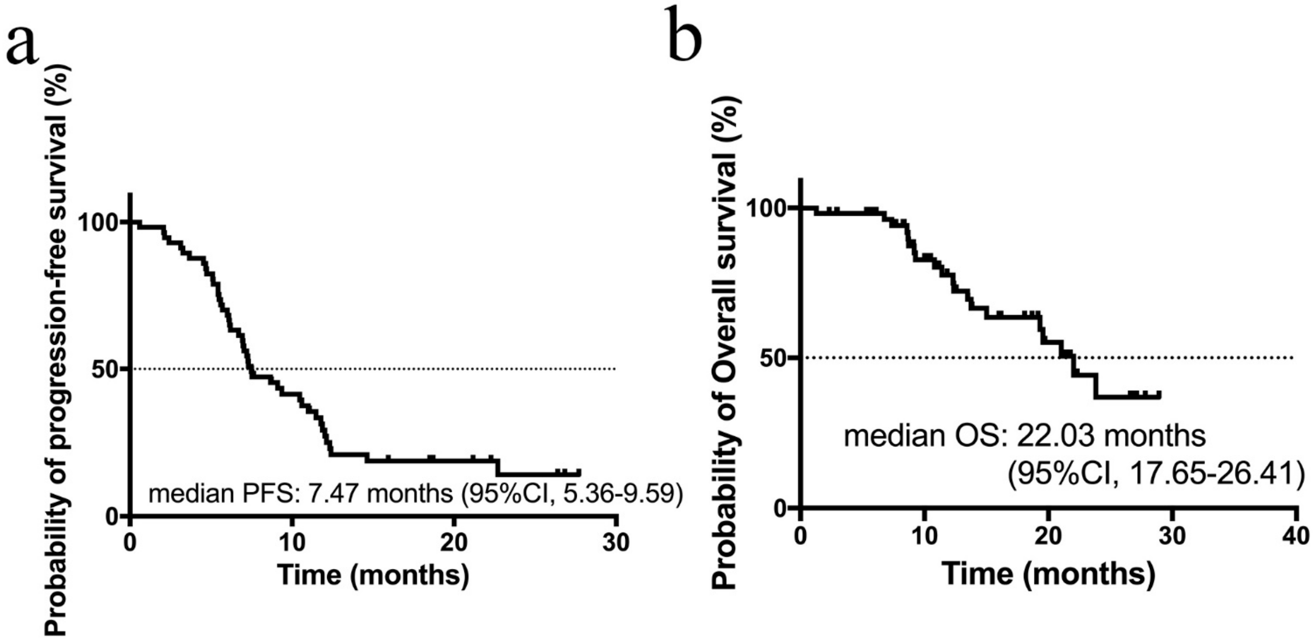

Figure S1. (a) Kaplan-Meier curve of progression free survival (PFS) of SCLC patients (N = 57). (b) Kaplan-Meier curve of overall survival (OS) of SCLC patients (N = 57).

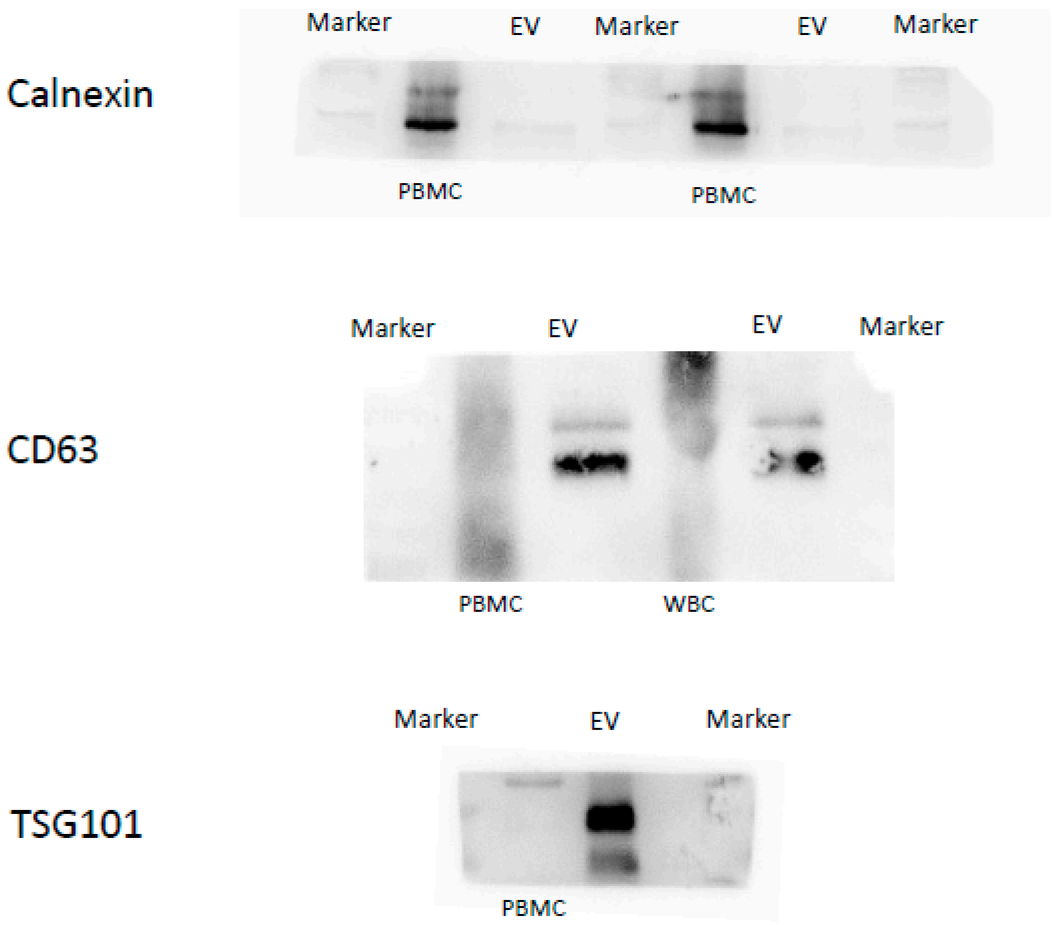

Figure S2. Original Western blot figures of Figure 1c.
